# Zinc Ion-Stabilized Aptamer-Targeted Black Phosphorus Nanosheets for Enhanced Photothermal/Chemotherapy Against Prostate Cancer

**DOI:** 10.3389/fbioe.2020.00769

**Published:** 2020-08-31

**Authors:** Li Gao, Ruobing Teng, Sen Zhang, Yun Zhou, Miaomiao Luo, Youqiang Fang, Lei Lei, Bo Ge

**Affiliations:** ^1^Department of Urology, Affiliated Hospital of Guilin Medical University, Guilin Medical University, Guilin, China; ^2^School of Pharmaceutical Sciences (Shenzhen), Sun Yat-sen University, Shenzhen, China; ^3^Department of Urology, The Third Affiliated Hospital of Sun Yat-sen University, Guangzhou, China; ^4^State Key Laboratory of Ophthalmology, Zhongshan Ophthalmic Center, Sun Yat-sen University, Guangzhou, China; ^5^Department of Urology, The Second Affiliated Hospital of Guilin Medical University, Guilin Medical University, Guilin, China

**Keywords:** black phosphorus, photothermal therapy, chemotherapy, zinc ion, targeted therapy

## Abstract

Prostate cancer is the second most common malignancy among men worldwide. However, conventional chemotherapy, such as taxane therapy, fails to exhibit efficient treatment for almost half of the patients. In this study, a nano-drug delivery system based on black phosphorus nanosheets (BP NSs) was developed, which was then employed as a multifunctional nanoplatform for targeted combinational chemo-photothermal therapy against prostate cancer. Zinc ion (Zn^2+^), which has been proven to be able to inhibit prostate cancer cell proliferation, was also introduced into this system. Zn^2+^ coordination could not only enhance the therapeutic effect of combined chemo-photothermal therapy, but also improve the intrinsic instability of BP NSs through the stabilization of its lone pair electrons. The *in vivo* study showed the outstanding performance of this system in targeted photothermal/chemotherapy of prostate cancer without side effect to normal organs.

## Introduction

Prostate cancer is the second most common malignancy among men around the world (Heidenreich et al., 2014). Early stage localized prostate cancer can be treated effectively by hormone and radiation therapy with less difficulty, however, the treatment of advanced-stage prostate cancer remains a significant challenge due to its frequent pathophysiological changes, epithelial-mesenchymal transition and drug resistance (Chen et al., 2018; Mollica et al., 2019; Wang et al., 2019c). Unfortunately, most patients with prostate cancer are already in late-stage at the time of consultation (Wang et al., 2019c; Teo et al., 2019). Chemotherapy is often employed for the treatment of advanced-stage aggressive and metastatic prostate cancer, nonetheless, chemotherapy, such as taxane therapy, fails to exhibit a good response for half of the patients (Li and Mahato, 2014, 2015). Research and development of new drugs is a potential option, however, the long time and significant investment of developing new drug molecules inevitably results in a high cost of therapy. Therefore, it is of great urgency to develop a high effective and relatively affordable method for the treatment of prostate cancer, especially late-stage prostate cancer.

Nowadays, with some novel therapeutic methods such as gene therapy, photodynamic therapy (PDT), and photothermal therapy (PTT) etc., emerging, the combination of two or more treatments has become a promising strategy to improve the therapeutic efficacy of chemotherapy (Cheng et al., 2017a; Guo et al., 2019). Due to the noninvasive, controllable and highly selective characteristics for cancer treatment, PTT has attracted wide attention for the past few decades (Yang et al., 2018; Cheng et al., 2019; Wang et al., 2019a). To date, various types of photothermal agents have been reported, including carbon-based nanomaterials, semiconductor nanoparticles, metal-based nanostructures, organic polymers and metal-organic frameworks (Li et al., 2015; Cheng et al., 2017c; Yin et al., 2017; Zhang et al., 2017; Liang et al., 2019).

As a novel 2D material, black phosphorus nanosheets (BP NSs) possess excellent extinction coefficient and high photothermal conversion efficacy, which enables BP NSs to be promising photothermal agents (Sun et al., 2015; Luo et al., 2019b). In addition, BP NSs completely meet the strict safety requirements in clinical use. The metabolism of BP is phosphate or phosphonate, which would not cause certain immune responses (Liu et al., 2019a; Luo et al., 2019a; Yang et al., 2019). Besides, compare with other 2D nanomaterials, such as Xenes, boron NSs, antimonene and MXene (Liu et al., 2017; Tao et al., 2019; Xue et al., 2019a; Tang et al., 2020), BP NSs possess large specific surface area due to a corrugated plane configuration, and thus can serve as an efficient delivery platform for a variety of different cargos, such as anticancer drugs, mental ions, targeting molecules, and so on, forming synergistic therapeutic systems.

However, although BP is the most stable allotrope of phosphorus, BP is susceptible to degradation upon exposure to ambient conditions (Liu et al., 2019b; Tao et al., 2019). BP is very reactive to water and oxygen, causing compositional and physical changes of BP (Favron et al., 2015; Ryder et al., 2016). The biomedical applications of BP are greatly limited owing to its instability under ambient environment. One P atom of BP is covalently bonded to another three single-layer P atoms, thereby exposing a pair of lone pair electrons (Ziletti et al., 2015). Such lone pair electrons would readily react with oxygen to form P_x_O_y_, which would be subsequently removed by water, resulting in the destruction of P network of BP NSs (Zhou et al., 2016; Liu et al., 2019a). Thus, it is supposed to be an effective strategy to mitigate oxidation of BP NSs under ambient environment if the lone pair electrons could be stabilized through occupation by other elements (Zhao et al., 2016; Guo et al., 2017). Herein, zinc ion (Zn^2+^) was employed to interact with BP to form Zn^2+^-modified BP NSs. The coordination of Zn^2+^ with the lone pair electrons of BP may impede the reaction between P and O_2_, thus ultimately improving the stability of BP.

More importantly, the organ with the highest level of Zn in human body is prostate. Zn can inhibit the activity of mitochondrial aconitase, which is of great significance in maintaining health and normal functions of prostate (Kelleher et al., 2011). However, Zn concentration dramatically diminishes during prostate malignancy (Kolenko et al., 2013; Xue et al., 2019b). Zn in prostate epithelial cells is present in the form of Zn^2+^. Although the specific mechanism remains to be further studied, it has been reported that an increase of Zn^2+^ level in prostate cancer cells can inhibit cell proliferation, invasion and metastasis, and induce its apoptosis as well via inhibiting some cellular signaling pathways (Uzzo et al., 2006; Yan et al., 2010; Chen et al., 2013). Therefore, it is expected that the introduction of Zn^2+^ in our system could not only improve the stability of BP, but also enhance the therapeutic effect of combined photothermal/chemotherapy against prostate cancer.

To improve the efficacy of the combined treatment in prostate cancer therapy, endowing such theraputic system with tumor targeting property is a promising strategy. Aptamers (Apts), a single-stranded RNA or DNA oligonucleotide, have been shown as an excellent targeting agent for efficient penetration into biological compartments with nonimmunogenicity (Li et al., 2017; Liu et al., 2018). Different aptamers can be produced through chemical synthesis at low cost. As one of the DNA Aptamers, AS1411 Apt is able to bind to nucleolin (NCL) with high specificity and affinity (Tao et al., 2016). NCL is a multifunctional protein overexpressed on the plasma membrane in a variety of solid tumors including prostate cancer, which has been widely recognized as an attractive tumor marker (Kim et al., 2012). Here, in order to improve the active tumor targeting ability, our system was further modified with NH_2_-PEG-Apt.

In this study, we constructed a multifunctional system based on BP NSs for Zn^2+^ enhanced combined chemo/photothermal treatment against prostate cancer. Apt modification allowed this platform to possess great tumor targeting property, increasing accumulation of the nanoparticles in tumor sites. Additionaly, zinc ion conjugation could also improve the stability of BPs.

## Materials and Methods

### Materials

The bulk BP was purchased from Smart-Elements (Vienna, Austria). MTT, 1-Methyl-2-pyrrolidinone (NMP), dimethyl sulfoxide (DMSO), tris-(2-carboxyethyl)-phosphine hydrochloride (TCEP) and 4′,6-diamidino-2-phenylindole (DAPI) were purchased from Sigma-Aldrich (St. Louis, MO, United States). Zinc acetate was obtained from Aladdin (Los Angeles, CA, United States). Methoxy-PEG_2k_-amine (NH_2_-PEG) and maleimide-PEG_2k_-amine (NH_2_-PEG-MAL) were provided by Shanghai Yare Biotech, Inc. (Shanghai, China). Doxorubicin hydrochloride (DOX) was bought from Dalian Meilun Biology Technology Co., Ltd. (Dalian, China). Dulbecco minimum essential medium (DMEM), streptomycin, penicillin, FBS, were purchased from Thermo Fisher Scientific (Waltham, MA, United States). Human prostate cancer cell (PC3) was obtained from Guilin Medical University (Guilin, Guangxi, China).

### Preparation of BP NSs

BP nanosheets (BP NSs) were synthesized via a modified liquid exfoliation method (Zeng et al., 2018). Briefly, 5 mg of bulk BP crystal powder was dispersed in 20 mL NMP solution, and then the mixture was subjected to probe sonication in an ice bath using a power of 700 W for 8 h (On/Off cycle: 2 s/4 s). The resulting brown suspension was centrifuged at 3000 rpm for 15 min to remove the unexfoliated bulk BP. The supernatant was collected gently and centrifuged for another 15 min at 10,000 rpm. The precipitate was collected and resuspended in NMP. The BP NSs were stored under 4°C for further experiments.

### Preparation of BP-P-Apt

20 OD of Apt-SH was dissolved in 1 mL tris buffer (pH = 7.4, 10 mM), followed by addition of 2 mg NH_2_-PEG-MAL and 40 μg TCEP. The mixture was stirred in the dark for 3 h to obtain NH_2_-PEG-Apt. Then, 2 mg BP NSs was added to the above solution. After probe sonication for 10 min and stirring for 5 h, BP-P-Apt was obtained by centrifugation for 15 min at 10,000 rpm and washed with deionized water for two times.

### Zinc Ion Conjugation

7.5 mg of zinc acetate was mixed with 5 mL BP-P-Apt nanosheets suspension (0.5 mg mL^–1^ BP) and subjected to probe sonication for 3 min. After stirring for 3 h, the mixture solution was centrifuged at 10,000 rpm for 15 min. The precipitate (Zn-BP-P-Apt) was collected and washed with deionized water.

### Drug Loading

Two milligram of Zn-BP-P-Apt was dispersed in 2 mL of DOX aqueous solution (1.5 mg mL^–1^) and then stirred for 6 h in the dark. After centrifugation (10,000 rpm, 15 min), the final product Zn-BP-P-Apt/D was separated and washed, and then freeze-dried for further use.

### Characterization of NSs

Transmission electron microscopy (TEM) images were acquired using FEI Tecnai G2 F30 transmission electron microscope. Atomic force microscopy (AFM) was performed on Bruker Diension Icon microscope. X-ray photoelectron spectroscopy (XPS) was performed using a Kratos Axis Ultra DLD spectrometer with Al Kα radiation (1486.6 eV photons, 150 W). The size and zeta potential of samples were measured on Malvern Mastersizer 2000 (Zetasizer Nano ZS90, Malvern Instruments Ltd., United Kingdom).

### pH/NIR Dual-Responsive DOX Release

The drug release experiments of Zn-BP-P-Apt/D were investigated in PBS with different pH values. For each study, 2mL of Zn-BP-P-Apt/D suspension (5 mg mL^–1^) was sealed in a dialysis bag (MWCO 3500, Shanghai Sangon, China). The dialysis bag was then immersed in 10mL PBS buffer medium (pH = 5.0 or 7.4) and shaken gently at 37°C (120 rpm). At predetermined time points, the Zn-BP-P-Apt/D suspension with pH 5.0 was irradiated by 808nm NIR laser (6min, 1.0W cm^–2^). At given time intervals, 0.5mL of the outside release medium was collected, and an equal volume of fresh medium was replenished to the old PBS. The cumulative amount of DOX released from NPs was measured using fluorescence spectrophotometry.

### *In vitro* Photothermal Effect

Photothermal performance of prepared NPs was evaluated via measuring temperature changes with an infrared thermal camera (Ti450, Fluke, United States). Zn-BP-P-Apt/D NPs with different concentrations from 50 to 200 μg mL^–1^ were irradiated with an 808 nm laser (Shanxi Kaisite Electronic Technology Co., Ltd., Xi’an, China) at power densities of 0.5, 1.0, and 2.0W cm^–2^ for 10min. To compare the photothermal effect of different NPs, BP, Zn-BP-P-Apt/D, and water were exposed under an 808nm laser for 10min with a power density of 1.0W cm^–2^.

### Stability Evaluation of BP NSs

To evaluate the influence of Zn^2+^ coordination on BP stability, bare BP and Zn-BP-P-Apt/D NSs with the same amount of BP concentration (100 μg mL^–1^) were dispersed in water and exposed to air for 6 days and then their photothermal properties were tested at predetermined time intervals. Then, bare BP and Zn-BP NSs were dispersed in water and exposed to air for 2 days. The morphology of these two samples were observed by ultra-depth three-dimensional microscope.

### Cell Culture and Cellular Uptake

Human prostate cancer cell (PC3) was cultured in high glucose DMEM medium containing 20% FBS, streptomycin (100μg mL^–1^), and penicillin (100 units mL^–1^). The cell culture condition at 37°C under 5% CO_2_.

PC3 cells (10^6^ cells per well) were seeded in a 20 mm glass-bottom Petri dish overnight. Then fresh medium containing DOX or DOX-loaded BP NSs at the concentration of 5 μg mL^–1^ DOX were added and incubated for 2 h. After that, cells were washed with PBS for three times, fixed with 4% (w/v) formaldehyde solution for 20 min, and stained with DAPI for 10 min, successively. Finally, the cells were observed on a confocal laser scanning microscope (CLSM, Olympus Fluoview FV-1000, Tokyo, Japan).

### *In vitro* Cytotoxicity Assay

To evaluate the cytotoxicity of zinc ion, PC3 cells (about 10^5^ cells per well) were allowed to culture in 96-well plates overnight. Afterward, the old DMEM was replaced by fresh culture medium containing different concentrations of zinc ion (1, 2.5, 5, 7.5, 10, 20, and 30 μg mL^–1^) and incubated for another 24, 48, and 72 h, respectively. The cell viability was determined by MTT assay. The percentage of cell viability was measured by comparison with the media alone group (negative control).

The evaluation of cytotoxicity of DOX on PC3 cells was performed using a similar procedure as described above except that the DOX concentration was 0.05, 0.1, 0.5, 1, 2.5, 5, and 10 μg mL^–1^.

The enhancement of zinc ion on chemotherapy of DOX was assessed using MTT assay. The concentration of zinc ion was 1 μg mL^–1^ and DOX concentration was from 0.05 to 10 μg mL^–1^. The culture time was 48 h.

To evaluate the combinational photothermal chemotherapy enhanced by zinc ion, PC3 cells at a density of 10^5^ cells per well were cultured in 96-well plates. After incubation overnight, fresh culture medium containing various samples with different concentrations (0.1, 0.5, 1, 2.5, and 5 μg DOX mL^–1^) were added to each well. For the NIR irradiation groups, cells were exposed to NIR laser at 1 W cm^–2^ for 10 min after addition of NPs for 4 h. Then the cells were incubated for another 48 h and the cell viabilities were calculated by MTT assay.

### *In vitro* Photothermal Therapy Study

PC3 cells were first seeded in a 96-well plate for 24 h. After that, the culture medium of each well was refreshed, and cells were incubated with BP, BP-P, and BP-P-Apt at different concentrations for 4 h at 37°C. The cells were subsequently subjected to 808 nm laser irradiation (1 W cm^–2^) for 10 min. The treated cells were incubated again for an additional 12 h. Finally, the cell viabilities were evaluated by MTT assay.

### Tumor Model Establishment

Female sever combined immunodeficient (SCID) mice were purchased from the Sun Yat-sen University Laboratory Animal Center. Animal experiments were performed following protocols approved by the Administrative Committee on Animal Research in Sun Yat-sen University. To develop the tumor model, PC3 cells (1 × 10^6^) in 100 μL PBS were subcutaneously injected into the right flank area of each mouse. Tumor sizes were measured with a digital vernier caliper every the other day. The tumor volume (V) was calculated by the equation: V = 0.5 × a × b^2^, where a and b represented length and width of the tumor, respectively.

### *In vivo* Infrared Thermal Imaging

When the volumes of the PC3 tumors reached about 500 mm^3^, the mice were injected with PBS, Zn-BP-P/D, and Zn-BP-P-Apt/D via the tail vein. After 24 h, the mice were anesthetized and the tumor sites were irradiated with a 808 nm NIR laser (1.5 W cm^–2^, 5 min). During the irradiation, an infrared thermal image camera was used to monitor the temperature changes and infrared thermographic maps.

### *In vivo* Biodistribution

Tumor bearing mice were administered intravenously with 100 μL DOX, Zn-BP-P/D, and Zn-BP-P-Apt/D (100 μL, 5 mg DOX mL^–1^), respectively. 3 or 24 h later, the mice were sacrificed, and the heart, liver, spleen, lung, kidney and tumor were collected. Subsequently, the distributions of DOX in these tissues were measured using the Maestro^TM^ Automated *In-Vivo* Imaging system (CRi Maestro^TM^, United States).

### *In vivo* Antitumor Therapy and Histochemistry Analysis

When the tumor volume reached 80mm^3^, PC3 tumor-bearing mice were randomly divided into 6 groups (*n* ≥ 5) and treated with (1) PBS, (2) DOX, (3) BP-P/D, (4) BP-P-Apt/D, (5) BP-P-Apt/D + NIR, and (6) Zn-BP-P-Apt/D + NIR (fixed DOX concentration at 5 mg kg^–1^, 100 μL). The injection was conducted every 4 days. The NIR groups were irradiated by 808 nm laser at power density of 1.0 W cm^–2^ for 5min after intravenous injection for 24h. Tumor volumes and body weights of the mice were monitored every 2 days. After 16 days of treatment, All the mice were euthanized. Tumors and main organs including heart, liver, spleen, lung and kidney were dissected, washed and used for histology analysis and TUNEL immunofluorescence staining.

## Results and Discussion

### Preparation of Zn-BP-P-Apt/D

**SCHEME 1:**
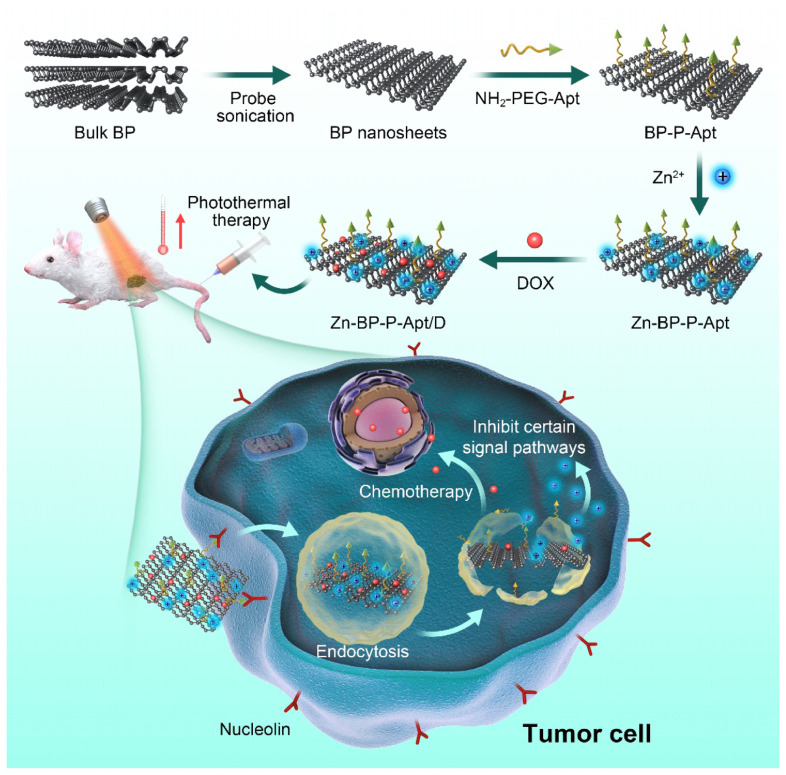
Schematic illustration for the fabrication process, and combined anticancer therapy of Zn-BP-P-Apt/D.

The synthetic process of Zn-BP-P-Apt/D nanoplatform was displayed in Scheme 1. The BP NSs used in this work were successfully prepared according to a modified liquid exfoliation technique from bulk BP. NH_2_-PEG-Apt was firstly modified on the surface of BP NSs via electrostatic adsorption to elevate targeting ability as well as the biocompatibility. Then, zinc ion was conjugated to BP NSs surface and the loading content of Zn^2+^ was about 12.8% (see Supplementary Figure S1). The conjugation of Zn^2+^ was expected to enhance the therapeutic effect of the combinational photothermal/chemotherapy. Meanwhile, the introduction of Zn^2+^ also contributed to improve the stability of BP NSs. After that, anticancer drug DOX was loaded for chemotherapy with loading content (LC) of 15.2% (Supplementary Figure S2).

### Characteristics of BP Based NSs

**FIGURE 1:**
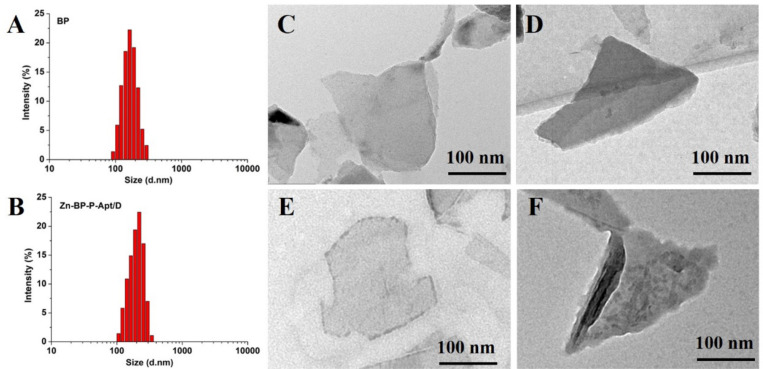
DLS size distribution of **(A)** BP NSs and **(B)** Zn-BP-P-Apt/D. TEM images of **(C)** BP sheets, **(D)** BP-P-Apt, **(E)** Zn-BP-P-Apt, and **(F)** Zn-BP-P-Apt/D.

TEM was utilized to characterize the morphology of BP and BP based nanosheets. As shown in Figures 1C–F, the size of bare BP and modified BP NSs were about 200–300nm, which was consisted with that from dynamic light scattering analysis (Figures 1A,B and Supplementary Figure S3). After introduction of Apt and Zn^2+^, the morphology of BP did not show any obvious difference (Figures 1D,E). In Figure 1F, as the drugs loaded on the BP NSs, a rougher surface could be obviously observed in TEM picture, indicating the successful loading of drugs. AFM image (Supplementary Figure S5) showed that the height of Zn-BP-P-Apt/D was about 5.1 nm.

**FIGURE 2:**
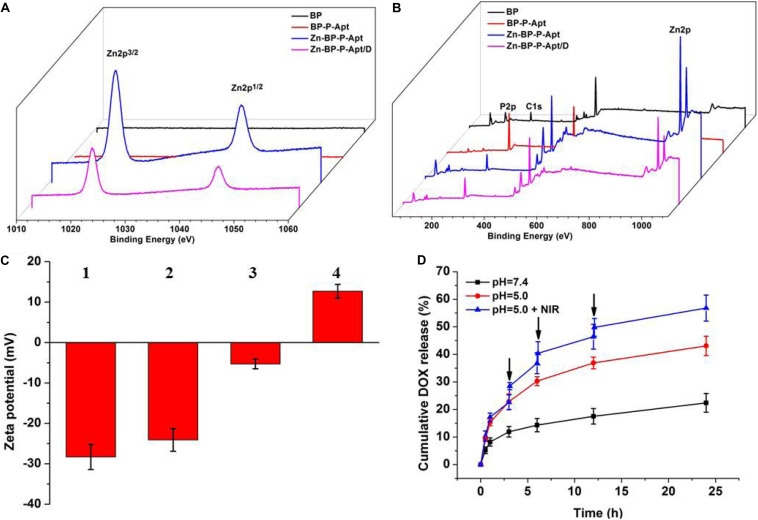
XPS spectra of BP, BP-P-Apt, Zn-BP-P-Apt and Zn-BP-P-Apt/D. **(A)** Narrow scan for Zn2p peaks **(B)** Survey of all tested peaks. Zeta potentials of BP-based NPs. **(C)** Zeta potentials of BP-based NPs. 1, 2, 3, and 4 represent BP, BP-P-Apt, Zn-BP-P-Apt, and Zn-BP-P-Apt/D, respectively. **(D)** Drug release profiles of Zn-BP-P-Apt/D at pH 7.4 and pH 5.0 with or without NIR irradiation, ↓: NIR irradiation for 10min.

As shown in Figure 2C, the original zeta potential of bare BP was around −28.3mV, and zeta potential subsequently increased to −24.1mV after introdution of NH_2_-PEG-Apt on the surface of BP. With the conjugation of Zn^2+^, zeta potential of PB-P-Apt-Zn changed to −5.3 mV. Lastly, it increased to 12.7mV after loading DOX.

The chemical composition of various NSs was examined by XPS (Figures 2A,B and Supplementary Figure S4). Figure 2A displays the Zn2p spectra of the four samples. The Zn2p peaks at 1045.3 and 1022.2 eV were observed from Zn-BP-P-Apt and Zn-BP-P-Apt/D, but no Zn2p peaks were detected from the bare BP and BP-P-Apt, proving the successful conjugation of zinc ion. Compared with BP-P-Apt-Zn, the Zn2p peak intensity of Zn-BP-P-Apt/D was weaker, which was due to the loading of DOX. Moreover, The P2p peak intensity of bare BP, BP-P-Apt, Zn-BP-P-Apt, and Zn-BP-P-Apt/D gradually decreased (Supplementary Figure S4A). This trend was because that there is no P element in PEG, Apt, Zn^2+^ and DOX. Therefore, this result suggested again the successful modification of the corresponding compounds.

### pH- and Temperature-Dependent Drug Release

As can be seen from Figure 2D, the cumulative amount of DOX released from Zn-BP-P-Apt/D within 24 h was about 43.1%. As a contrast, only 22.4% of DOX was released during the same period at pH 7.4. This exhibited that the acidic environment would accelerate the release of loaded DOX. It might be owing to the fact that acidic environment could increase the water solubility of DOX, thus leading to a faster release of drug. This release behavior was very meaningful. DOX would not be released from Zn-BP-P-Apt/D when the NSs were circulated in the neutral environment of blood (pH = 7.4). Once these NSs entered tumor region, the acidic environment of tumor would accelerate the release of anticancer drugs. Stimulated by 3 cycles of 808nm laser on/off treatment, DOX cumulative rate at pH 5.0 increased to 56.8% after 24 h, proving the photothermal-induced drug release behavior. These results suggested pH- and temperature-responsive drug release of Zn-BP-P-Apt/D, which could not only reduce the side effects of drugs but also improve its utilization rate. Besides, the drug release profiles of Zn-BP-P-Apt/D at pH6.5 and 6.0 were also tested (Supplementary Figure S6).

### Photothermal Properties of Different NSs

**FIGURE 3:**
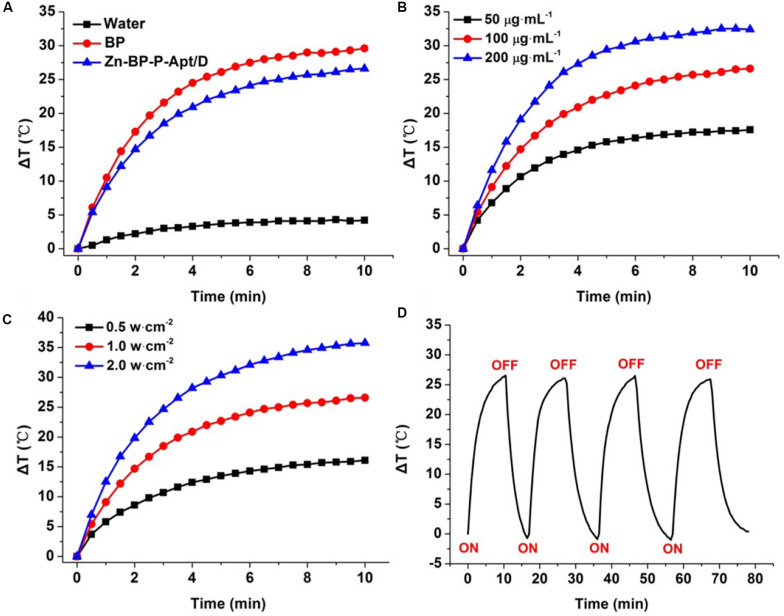
**(A)** Changes in temperature of pure water, BP NSs and Zn-BP-P-Apt/D. **(B)** Temperature elevation curves of Zn-BP-P-Apt/D suspension with different concentrations at a power density of 1.0 W cm^–2^. **(C)** Temperature change curves of Zn-BP-P-Apt/D suspension with the concentration of 100μg mL^–1^ at different laser powers. **(D)** Heating of a suspension of the Zn-BP-P-Apt/D in water for four laser on/off cycles with an 808 nm NIR laser at power density of 1.0 W cm^–2^.

To examine the photothermal properties of different NSs, water, aqueous solutions of BP and Zn-BP-P-Apt/D were exposed to laser irradiation of 808nm. The temperature changes were monitored and quantified by an infrared thermal imaging camera (Figure 3). According to the equations in supporting information, the photothermal conversion efficiency of BP NSs, Zn-BP-P-Apt, and Zn-BP-P-Apt/D was calculated to be 29.6, 27.1, and 24.3% separately. As shown in Figure 3A, the temperature of BP solution (100μg mL^–1^) increased by ∼29.6°C after irradiation (1 W cm^–2^) for 10min, while the pure water was hardly heated by the irradiation, indicating that BP could effectively convert NIR light into thermal energy. The temperature rise of Zn-BP-P-Apt/D was about 3°C lower than BP (ΔT = 26.6°C). However, this temperature change was higher enough to induce irreversible cell apoptosis owing to hyperthermia (Wust et al., 2002). We next investigated the impact of power density of NIR laser on the Zn-BP-P-Apt/D-induced hyperthermia (Figure 3C). A dispersion of Zn-BP-P-Apt/D at 100μg BP mL^–1^ was irradiated with 808 nm laser with different laser power density, implying that the temperature change of the dispersion could still reach about 16.1°C even the power density was as low as 0.5 W cm^–2^. Moreover, Zn-BP-P-Apt/D exhibited a concentration-dependent photothermal property (Figure 3B). As presented in Figure 3D, after irradiation with NIR laser light for 4 cycles, the process of temperature changes did not show any significant change, suggesting satisfactory photostability of Zn-BP-P-Apt/D.

### Stability Evaluation

**FIGURE 4:**
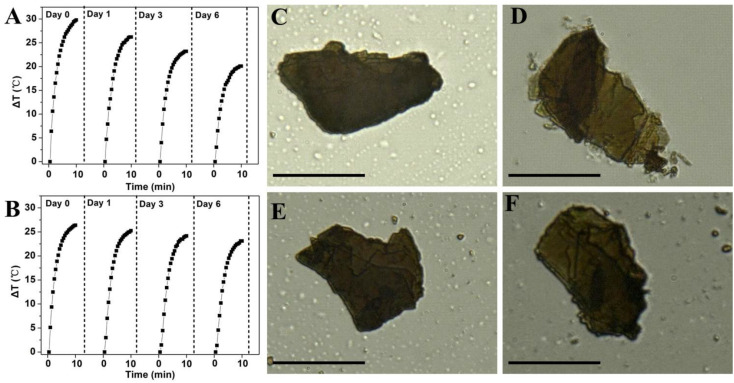
Photothermal heating curves of **(A)** bare BP and **(B)** Zn-BP-P-Apt/D dispersed in air-exposed water for 0, 1, 3, and 6 days. The 808nm laser was used as the irradiation source at the power density of 1 W cm^–2^. Optical images of bare BP on Si/SiO_2_ after exposure to the humid air at room temperature for **(C)** 0 and **(D)** 48 h, images of bare Zn-BP after **(E)** 0 and **(F)** 48 h (Scale bar = 10 μm).

To study whether coordination with Zn^2+^ could improve the stability of BP, the photothermal performance of bare BP and Zn-BP-P-Apt/D (100μg BP mL^–1^) in air-exposed water was tested (Figures 4A,B). The temperature of bare BP rose by about 29.8°C within 10min, but irradiation elevated the temperature by only 20°C after 6 days, so the photothermal performance of BP was quickly attenuated due to its degradation. By contrast, Zn-BP-P-Apt/D was obviously more photothermally stable. The temperature rise only changed about 3.3°C (from 26.4to 23.1°C) after 6 days. Therefore, coordination with Zn^2+^ could considerably stabilize BP.

Next, we visually observed the stability of BP and Zn-BP-P-Apt/D by using ultra-depth three-dimensional microscope (Figures 4C–F). Being kept in air at room temperature with 95% humidity for 48h, the degradation of bare BP could be evidently noted, especially at the edges. On the contrary, the surface of Zn-BP-P-Apt/D NSs hardly changed, proving the robust stability of Zn-BP-P-Apt/D. These results altogether directly demonstrated that the coordination between zinc ion and BP NSs could effectively prohibit the BP oxidation in humid air, thus improving its stability.

### Cellular Uptake

**FIGURE 5:**
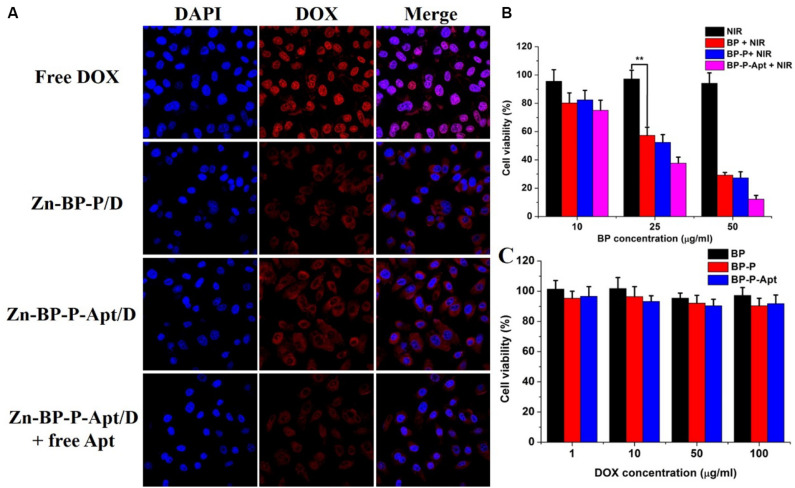
**(A)** Laser scanning confocal microscopy images of PC3cells treated with DOX, Zn-BP-PEG/D, Zn-BP-P-Apt/D, Zn-BP-P-Apt/D with free Apt added, and the incubation time was 2 h. **(B)** The cell viabilities of PC3 cells incubated with various concentrations of BP, BP-PEG, and BP-P-Apt with NIR laser irradiation. **(C)** *In vitro* cytotoxicity of BP, BP-P, and BP-P-Apt tested by MTT assay (808 nm, 1 W cm^–2^, 10 min, **P < 0.01).

It was reported that AS1411 aptamers could bind to a variety of tumor cells (Tao et al., 2016). To confirm this, the cellular uptake of DOX or DOX loaded NSs against PC3 cells was evaluated by CLSM. As shown in Figure 5A, compared with Zn-BP-P/D group, Zn-BP-P-Apt/D group exhibited a stronger fluorescent signal, implying that aptamer modified NPs were able to bind to PC3 cells efficiently. To further verify the tumor targeting ability of aptamers, an excessive amount of free AS1411 aptamers was added in Zn-BP-P-Apt/D group. After incubation together for 2 h, the red fluorescence intensity dramatically decreased. The reason for this might be that a large amount of free AS1411 aptamers bound to nucleolin (Apt receptors) on the plasma membrane, thus inhibiting the binding between Zn-BP-P-Apt/D and nucleolin to a large extent.

Interestingly, we found than free DOX group showed the strongest red fluorescent intensity, even better than Zn-BP-P-Apt/D. This might be ascribed to the fact that DOX could penetrate through the plasma membrane and nuclear membrane freely and quickly via passive diffusion effect due to its small molecule size (Cheng et al., 2017b). However, the *in vivo* microenvironment is way more complicated, owing to the sustained release and active tumor targeting effect, DOX-loaded NSs can improve the *in vivo* biocompatibility and biodistribution of anticancer drug, thus enhancing its therapeutic effect.

### *In vitro* Photothermal Therapy

To test the photothermal cytotoxicity of different BP NSs based nanomaterials *in vitro*, MTT assay was performed on PC3 cells. As shown in Figure 5B, BP, BP-P and BP-P-Apt displayed a concentration-dependent photothermal effect. As expected, due to active tumor targeting ability, Apt modified BP NSs (BP-P-Apt) showed the highest photothermal cytotoxicity and about 87.8% of PC3 cells were killed at the BP-P-Apt concentration of 50 μg mL^–1^, which was much higher than that of BP (70.8%) and BP-P (72.7%) groups at the same concentration. In contrast, NIR irradiation alone showed negligible cytotoxicity to PC3 cells.

### *In vitro* Cytotoxicity

**FIGURE 6:**
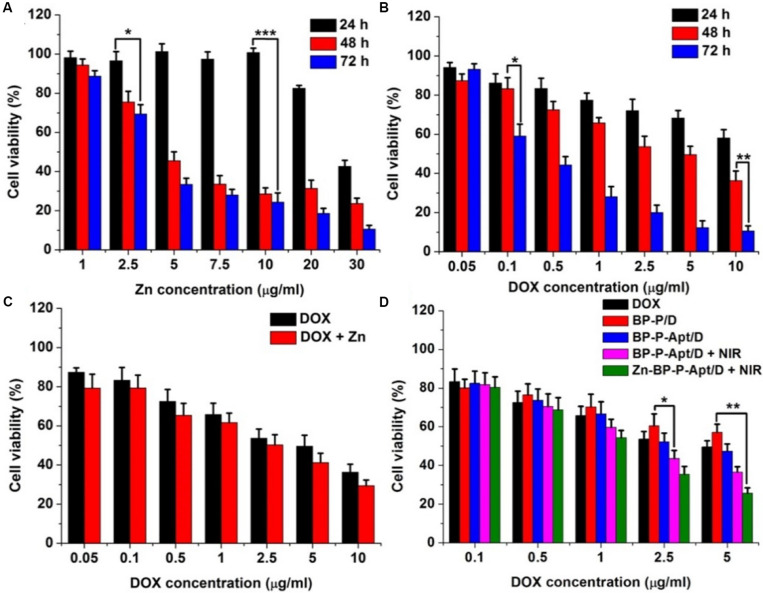
**(A)** Relative cell viability of PC3 cells after incubation with different concentrations of Zn^2+^ for 24, 48, and 72 h. **(B)** Cell viability of PC3 cells after incubation with DOX at different concentrations for 24, 48, and 72 h. **(C)** Cell viability of PC3 cells treated with DOX or DOX + Zn^2+^ (Zn^2+^ concentration was 1 μg mL^–1^) at various concentrations of DOX for 48 h. **(D)** Cell viability of PC3 cells treated with different samples with or without NIR laser irradiation at various concentrations of DOX for 48 h. (*p < 0.05, **p < 0.01, ***p < 0.001).

To access the cytotoxicity of BP based NSs to PC3 cells, MTT assay was employed. As displayed in Figure 5C, BP, BP-P and BP-P-Apt NSs exerted negligible cytotoxicity. For example, PC3 cells treated with BP-P-Apt still had about 91.8% cell viability even at a concentration of 100μg mL^–1^ after 48 h, confirming the excellent biocompatibility of the bare NSs. And the toxicity of Zn^2+^ to PC3 cells showed a time and dosage dependent manner. Zn^2+^ began to exhibit obvious cytotoxicity to PC3 cells after 48 or 72 h treatment when its concentration is more than 5 μg mL^–1^ (Figure 6A). The cytotoxicity of DOX to PC3 cells was also studied (Figure 6B). Figure 6C shows the combined cytotoxicity of Zn^2+^ and DOX to PC3 cells (at a fixed Zn^2+^ concentration of 1μg mL^–1^). It could be observed that zinc ion could enhance the *in vitro* theraputic efficacy of DOX to PC3 cells to a certain extent for all tested DOX concentrations.

Next, we investigated the combined cytotoxicity of zinc ion, DOX and photothermal therapy (Figure 6D). BP NSs loaded with DOX but without Apt and Zn^2+^ (denoted as BP-P/D) showed a moderate cytotoxicity, with about 57.1% cell viability at 5μg DOX mL^–1^ after 48 h. In contrast, after introduction of Apt, BP-P-Apt/D exhibited a stronger toxicity to PC3 cells due to active tumor targeting ability of Apt. After irradiation by NIR, BP-P-Apt/D showed a better toxicity for tumor cells killing in comparison with other formulas without NIR irradiation, denoting that the combined chemo/photothermal therapy could exert a better therapeutic effect than chemotherapy alone. What’s more, after Zn^2+^ conjugation, Zn-BP-P-Apt/D + NIR group showed the best tumor cells killing effect, implying the importance of synergistic actions of zinc ion enhanced combinational chemo/photothermal therapy for prostate cancer.

### *In vivo* Photothermal Imaging

**FIGURE 7:**
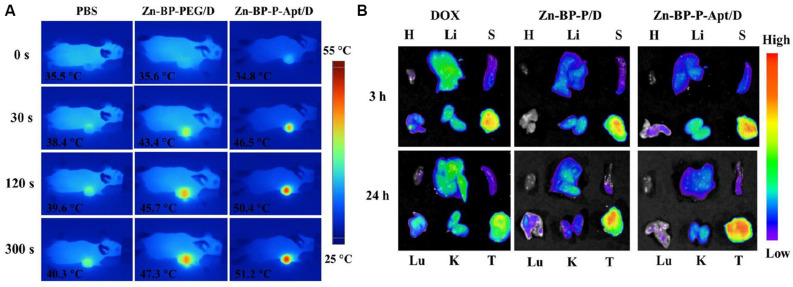
**(A)** *In vivo* IR thermal images of PC3 tumor-bearing mice treated with 808nm laser for 300 s. **(B)** *Ex vivo* fluorescent images of major organs and tumors after mice were injected with free DOX, Zn-BP-P/D, and Zn-BP-P-Apt/D at 3 and 24 h. H, Li, S, Lu, K, and T represent heart, liver, spleen, lung, kidney, and tumor, respectively.

IR thermal imaging of PBS and BP based NSs *in vivo* was investigated under irradiation with 808 nm laser (1.5 W cm^–2^) for 5 min after 24 h post injection. As presented in Figure 7A and Supplementary Figure S7, the tumoral temperature of PBS treated group rose slightly after 5 min laser irradiation. Differently, in the group treated with Zn-BP-P/D, hyperthermia was quickly generated in tumor region and reached up to around 47.3°C. Zn-BP-P-Apt/D showed a better performance than Zn-BP-P/D and the temperature rose to about 51.2°C within 5 min, which was high enough to kill tumor cells. These results demonstrated that Zn-BP-P-Apt/D could efficiently accumulate in tumor sites and act as superb photothermal agents to produce hyperthermia *in vivo* to effectively kill tumor cells.

### *In vivo* Biodistribution

The *in vivo* biodistribution of DOX and DOX loaded NSs were investigated in nude mice bearing PC3 tumors after tail vail injection. The fluorescence intensity of DOX was captured at 3 or 24h post injection (Figure 7B). After 3 h, DOX in Zn-BP-P/D and Zn-BP-P-Apt/D NSs was mainly distributed to tumor, while a relatively strong fluorescence intensity of DOX could be detected not only in tumor but also in liver for free DOX group. At 24h post injection. DOX intensity became weaker in tumor sites for free DOX group, implying its short retention time in blood and tissues. In contrast, a much stronger fluorescence signals of DOX in tumor were observed in both DOX loaded BP groups after 24 h. As expected, Zn-BP-P-Apt/D exhibited the strongest DOX signal in tumor, indicating an excellent tumor targeting ability. Overall, the DOX signal distribution for BP based NSs, especially for Zn-BP-P-Apt/D, revealed a predominant accumulation in tumor, a pattern expected for nanoparticle biodistribution.

### *In vivo* Therapeutic Effect

**FIGURE 8:**
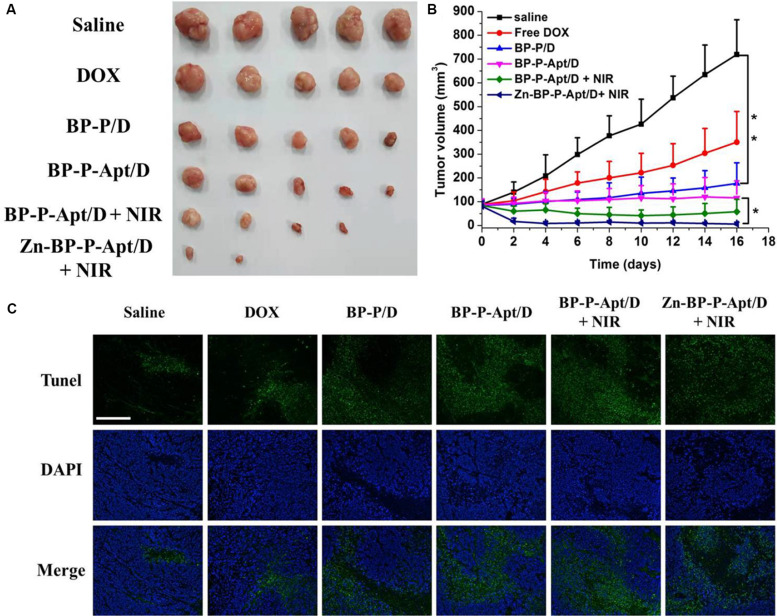
**(A)** Pictures of the tumors extracted from mice at day 16. **(B)** Curves of tumor growth after different treatments. **(C)** TUNEL fluorescence staining of tumor sections at the end of the *in vivo* antitumor experiment *p < 0.05, **P < 0.01). (Scale bar = 200 μm).

Motivated by the *in vitro* inspiring results, *in vivo* Zn^2+^ enhanced chemo/photothermal therapy was investigated. As shown in Figures 8A,B, a rapid growth of tumors was observed in PBS treated group during the 16-day treatment period. The tumor volume of the mice injected with free DOX could be partly but not significantly reduced in comparison with that of the control group, revealing that this dosage of administered DOX was not adequate to effectively kill tumor cells. By contrast, efficient anti-tumor effects were noticed in BP-P/D and BP-P-Apt/D groups compared with free DOX. This is attributed to more accumulation of DOX in tumor sites due to EPR effects (Cheng et al., 2017d; Wang et al., 2019b). Besides, BP-P-Apt/D showed a more effective tumor ablation effect than BP-P/D, implying a good tumor targeting ability of Apt. PC3 tumors were further inhibited in BP-P-Apt/D group after photothermal treatment. Inspiringly, the tumor growth curve in Zn-BP-P-Apt/D + NIR group revealed the lowest growth rate, and three mice in this group were completely cured after 16 days. This result proved the most excellent tumor inhibition effect of Zn-BP-P-Apt/D + NIR, which was ascribed to the Zn^2+^ enhanced combinational chemo/photothermal therapy.

The anticancer efficiency of Zn-BP-P-Apt/D is further analyzed by a terminal deoxynucleotidyl transferase-mediated deoxyuridine triphosphate nick end-labeling (TUNEL) assay, which is generally employed to examine the intratumoral levels of apoptosis. As displayed in Figure 8C, few TUNEL-positive cells (green color) were observed from the PBS, and pure DOX groups, while significant TUNEL-positive apoptotic cells could be observed from the Zn-BP-P-Apt/D NSs group.

The potential toxicity of different BP based NSs was studied. During the treatment time, there was no significant decrease in body weight of mice (Supplementary Figure S8), suggesting little side effects of the NSs. Moreover, as shown in Supplementary Figure S10, histological evaluation of major organs stained with hematoxylin and eosin (H&E) displayed no obvious inflammatory lesion or organ damage in all major organs after the treatment. This again demonstrated the good biocompatibility of NSs. Therefore, the as-prepared Zn-BP-P-Apt/D NSs showed a great potential for Zn^2+^ enhanced dual-modal cancer therapy.

## Conclusion

In summary, a tumor-targeting nano-drug system (Zn-BP-P-Apt/D NSs) was successfully developed for combined chemo-photothermal therapy against prostate cancer. The drug release experiment showed a pH- and NIR irradiation-responsive drug release behavior. Cytotoxicity assay indicated that Zn^2+^ itself could inhibit the proliferation of prostate cancer cells to some extent. Meanwhile, the photothermal/chemotherapy efficiency was further enhanced through introduction of Zn^2+^ into this multifunctional nanoplatform. Besides, Zn^2+^ coordination improved the stability of BP NSs, which is of great significance to slow down the degradation of its photothermal performance. Moreover, modification of PEG-Apt enabled extended blood circulation time and targeted accumulation at tumor sites. Both *in vitro* and *in vivo* anti-tumor assays demonstrated the excellent therapeutic efficacy of this nanodrug system for prostate cancer therapy.

## Data Availability Statement

All datasets generated for this study are included in the article/Supplementary Material, further inquiries can be directed to the corresponding authors.

## Ethics Statement

The animal study was reviewed and approved by the Administrative Committee on Animal Research in Sun Yat-sen University.

## Author Contributions

All authors listed have made a substantial, direct and intellectual contribution to the work, and approved it for publication.

## Conflict of Interest

The authors declare that the research was conducted in the absence of any commercial or financial relationships that could be construed as a potential conflict of interest.
