# Supplementary material for: Zinc Ion-Stabilized Aptamer-Targeted Black Phosphorus Nanosheets for Enhanced Photothermal/Chemotherapy Against Prostate Cancer
Source: Front Bioeng Biotechnol. 2020 Aug 31;8:769. doi: 10.3389/fbioe.2020.00769 (PMC7487335; doi:10.3389/fbioe.2020.00769)
Supplement: Supplementary file 1 [file Data_Sheet_1.docx]

**Supporting Information**

**Zinc Ion-Stabilized Aptamer-Targeted Black Phosphorus Nanosheets for Enhanced Photothermal/Chemotherapy against Prostate Cancer**

*Li Gao**^a, #^,Ruobing Teng ^a, #^, Sen Zhang^a^, Yun Zhou^b^, Miaomiao Luo^b^, Youqiang Fang^c^, Lei Lei^d,^ ^*^, Bo Ge ^a,e,^**^*^*

*^a^ Affiliated Hospital of* *Guilin Medical University, Guilin, Guangxi, 541001, China*

*^b^ School of Pharmaceutical Sciences (Shenzhen), Sun Yat-Sen University, Shenzhen 518107, China
^c^ Department of Urology, the Third Affiliated Hospital of Sun Yat-Sen University, Guangzhou, 510630, China*

*^d^ State Key Laboratory of Ophthalmology, Zhongshan Ophthalmic Center, Sun Yat-sen University, 7 Jinshui Road, Guangzhou 510060, China**^e^The Second Affiliated Hospital of* *Guilin Medical University, Guilin, Guangxi, 541199, China*

# These authors contributed equally to this work.

^*^ Corresponding author. Bo Ge, [ge1123@sina.com](mailto:ge1123@sina.com); Lei Lei, [leilei25@mail.sysu.edu.cn](mailto:leilei25@mail.sysu.edu.cn)

**Methods**

**Photothermal Conversion Efficiency Calculation.**

The photothermal conversion efficiencies of various NSs were calculated in detail as follows (here BP NSs were employed as an example):

The extinction coefficiency of BP NSs was determined according to the Beer-Lambert law:

$\varepsilon_{808}=\frac{A_{808}}{CL}$ (1)

where ε is the extinction coefficient, *A_808_* is the absorption at a wavelength of 808 nm of BP NSs, L is the path length (cm), and *C* (g L^-1^ ) is the concentration of the BP NSs.

The total energy balance for this system can be concluded as:

$\sum_{i} m_{i}C_{p,i}\frac{dT}{dt}=Q_{BP NSs}+Q_{s}-Q_{loss}$ (2)

where *C_p_* and m are the heat capacity and mass of the solvent (water), T is the solution temperature respectively.

$Q_{BP NSs}$ is the photothermal energy from BP NSs under irradiation:

$Q_{BP NSs}=I(1-{10}^{-A_{808}})\eta$ (3)

where I is the laser power, *A808* means the absorbance of BP NSs at the irradiation of 808 nm laser, and η is the photothermal conversion efficiency.

*Q_s_* is the heat which is absorbed by the water.

*Q_loss_* is the thermal energy trasnfering to the environment:

$Q_{loss}=hA\Delta T$ (4)

where h is the heat transfer coefficient, ΔT is the temperature change, and A is the surface area of the container.

At the maximum steady-state temperature, the heat input and the heat output are equal, that is:

$Q_{BP NSs}+Q_{s}=Q_{loss}=hA\Delta T_{max}$ (5)

*ΔT_max_* is the temperature change at the maximum steady-state temperature. According

to the Eq.3 and Eq.5, the *η* of BP NSs can be determined as:
$\eta=\frac{hA\Delta T_{max}-Q_{s}}{I(1-{10}^{-A_{808}})}$ (6)

For the sake of the value of *hA*, the ratio of *ΔT* to *ΔT_max_*, *θ*, was introduced :

$\theta=\frac{\Delta T}{{\Delta T}_{max}}$ (7)

Substituting Eq.7 into Eq.2 and rearranging Eq.2:

$\frac{d\theta}{\mathrm{dt}}=\frac{hA}{\sum_{i} m_{i}C_{p,i}}\left[ \frac{Q_{BP NSs}+Q_{s}}{hA\Delta T_{max}}-\theta\right]$ (8)

When the laser was off, Q_NSs_+Q_s_ = 0, so Eq.8 changed to:

$dt=-\frac{\sum_{i} m_{i}C_{p,i}}{hA}\frac{d\theta}{\theta}$ (9)

Integrating Eq.8 gives the expression:

$t=-\frac{\sum_{i} m_{i}C_{p,i}}{hA}\theta$ (10)

Thus, the *η* of BP NSs can be calculated through determining *hA* and substituting to Eq. 6.


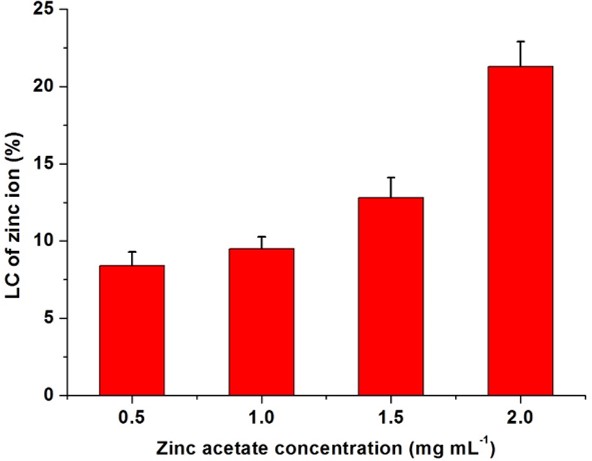


**Figure S1.** The Zn^2+^ loading capacity of BP-P-Apt under various Zinc acetate concentrations, LC = loading content, n=3


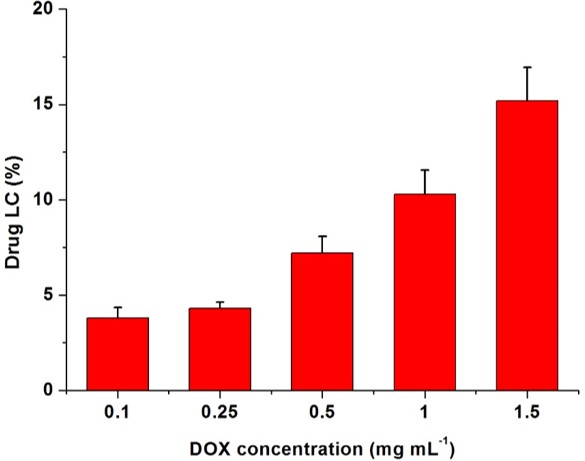


**Figure S2.** The DOX loading capacity of Zn-BP-P-Apt under various DOX concentrations, LC = loading content, n=3.


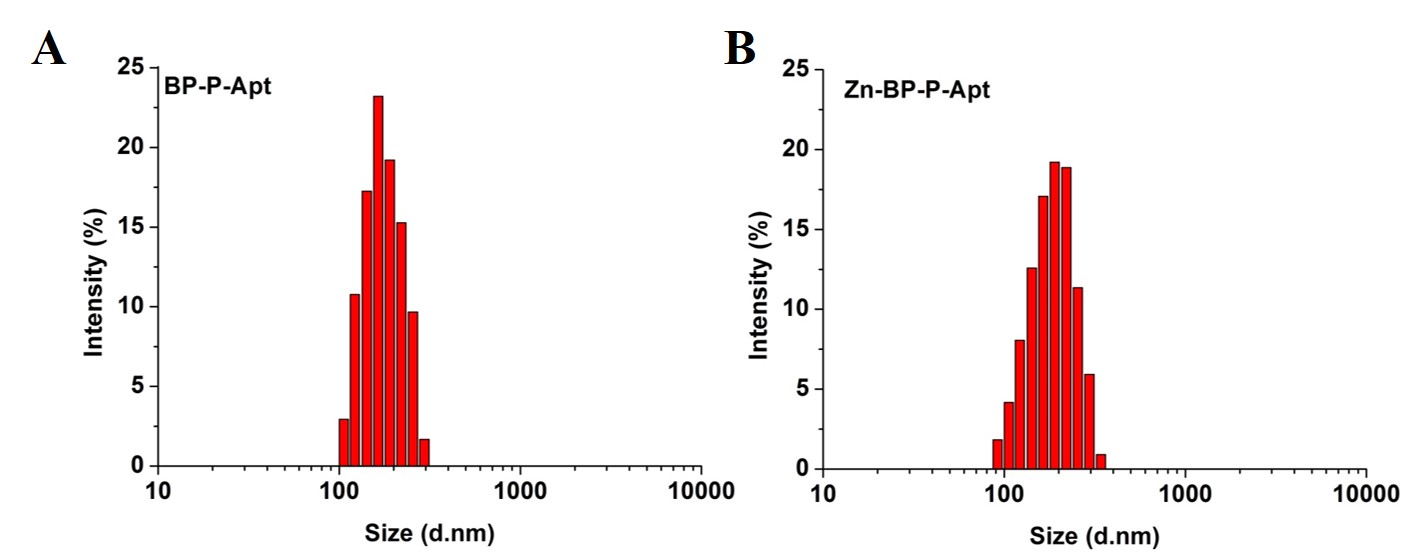


**Figure S3.** DLS size distribution of BP-P-Apt and Zn-BP-P-Apt.


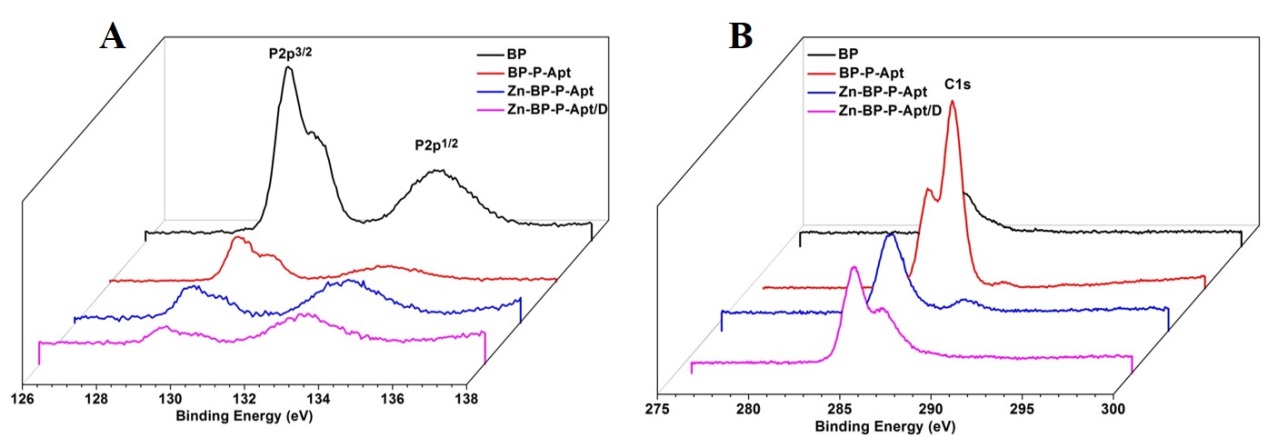


**Figure S4.** XPS spectra of BP, BP-P-Apt, Zn-BP-P-Apt and Zn-BP-P-Apt/D. (A) Narrow scan for P2p peaks, (B) Narrow scan for C1s peaks.


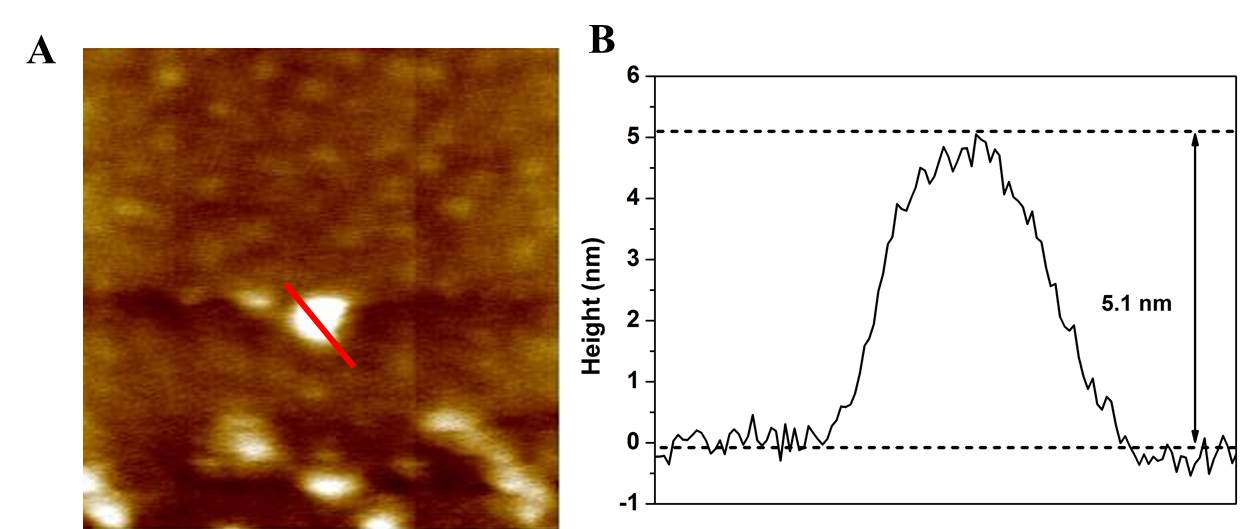


**Figure S5.** (A) AFM image of Zn-BP-P-Apt/D. (B) Height profiles along the red lines in (A).


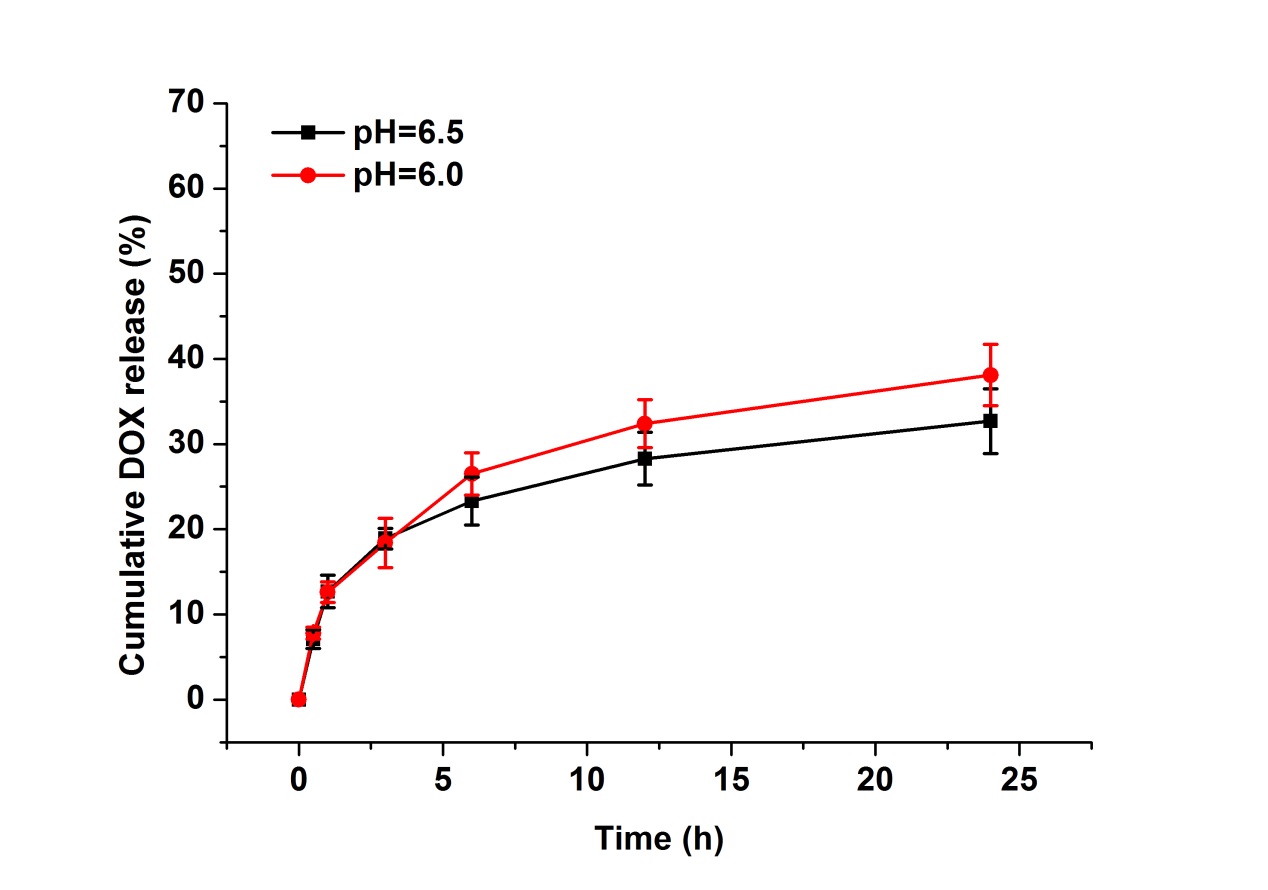


**Figure S6.** Drug release profiles of Zn-BP-P-Apt/D at pH 6.5 and pH 6.0.


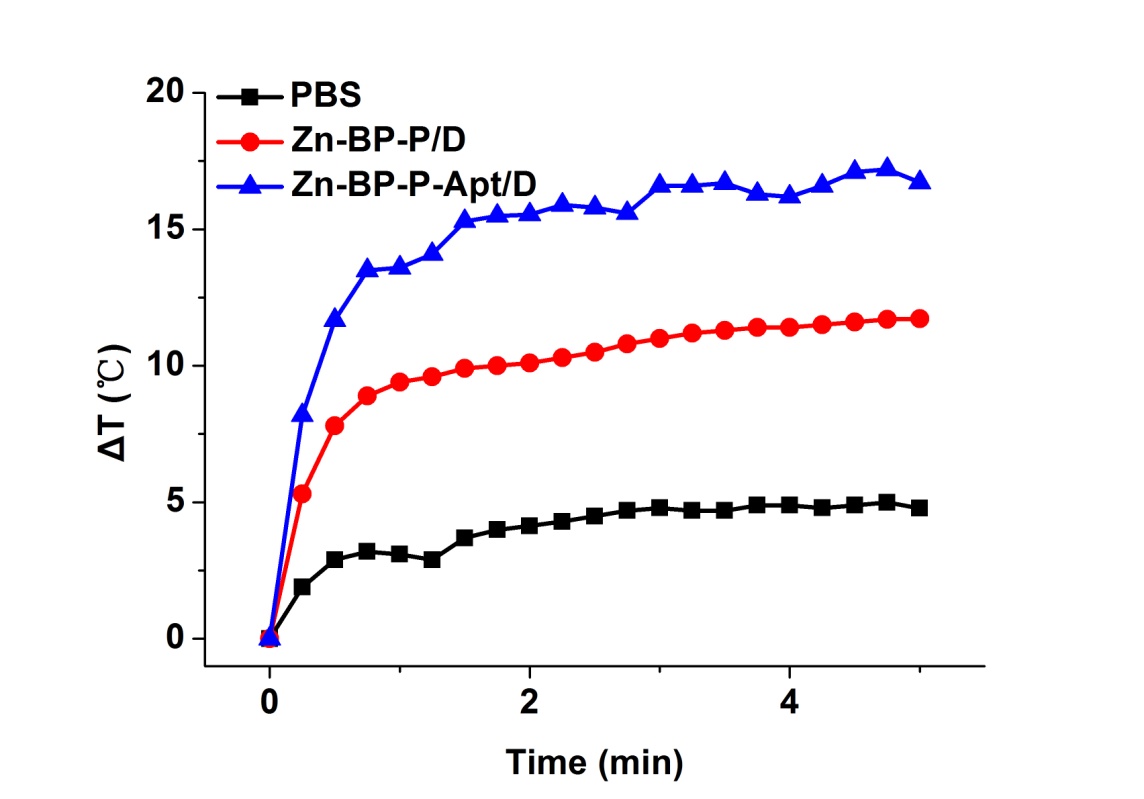


**Figure S7**. Time-dependent temperature increase of PC3 tumor-bearing mice recorded by an IR camera under 808 nm laser (1.5 W cm^-2^).


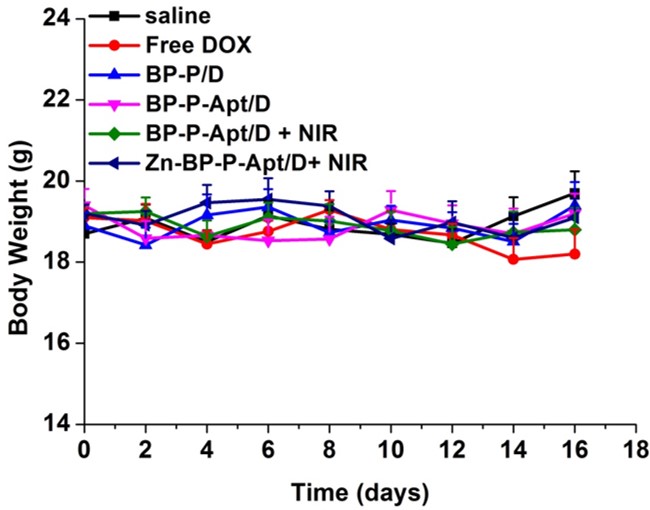


**Figure S8.** Body weights of the mice during evaluation period.


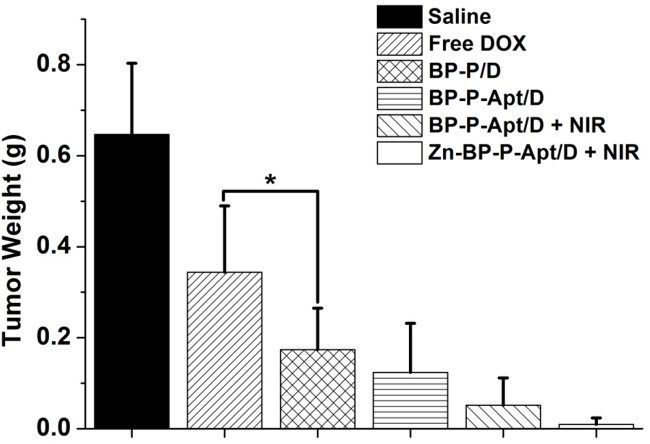


**Figure S9.** Tumor weight of each group taken out from the sacrificed mice at the end point of research (*p<0.05).


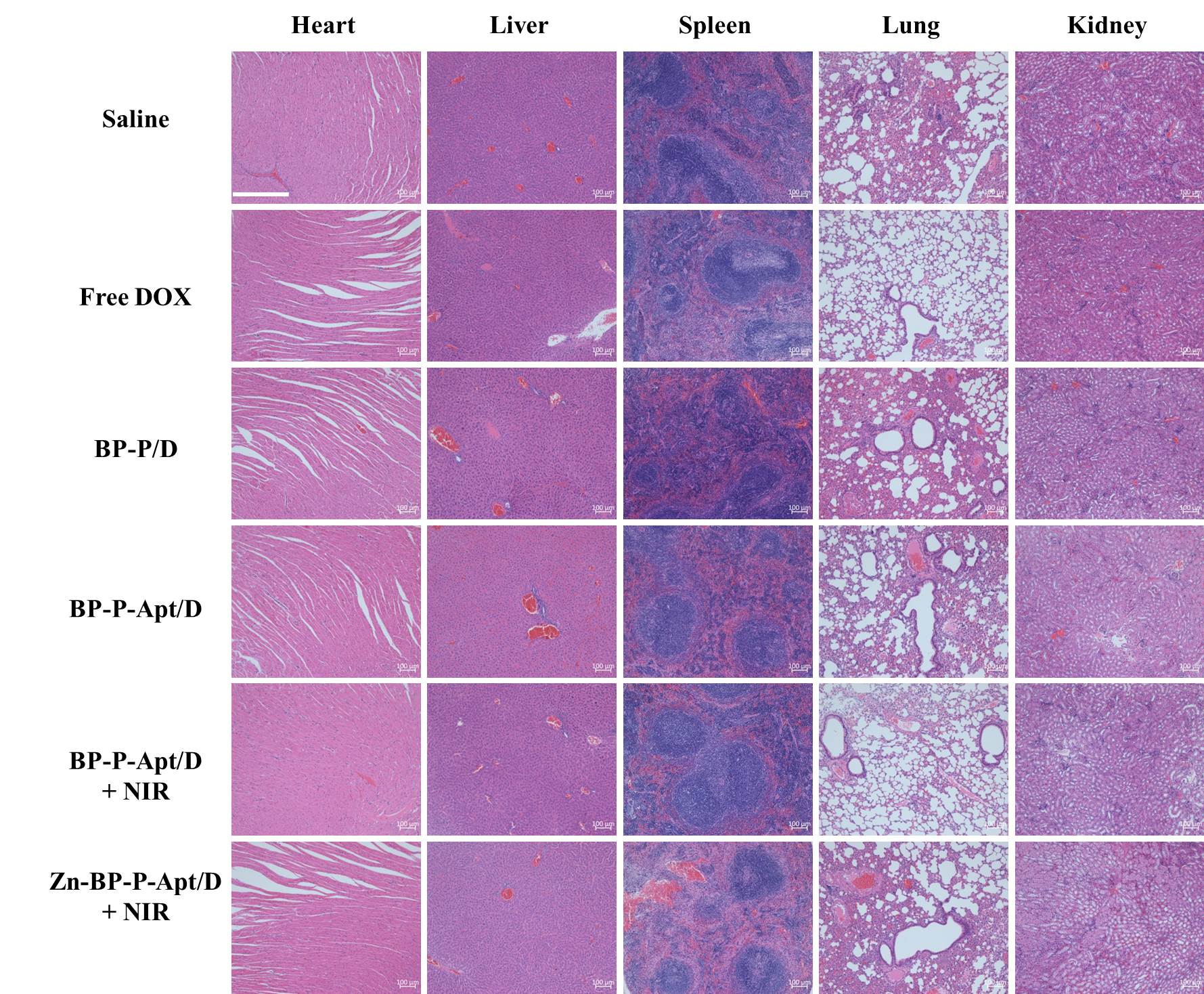


**Figure S10.** *In vivo* toxicity study. H&E-stained tissue sections of major organs of PC3 tumor-bearing mice (heart, liver, spleen, lung and kidney) after 16 days combined treatment. Saline-treated mice were used as the control. (Scale bar = 400 μm)
